# Loss of Grainy Head-Like 1 Is Associated with Disruption of the Epidermal Barrier and Squamous Cell Carcinoma of the Skin

**DOI:** 10.1371/journal.pone.0089247

**Published:** 2014-02-20

**Authors:** Michal Mlacki, Charbel Darido, Stephen M. Jane, Tomasz Wilanowski

**Affiliations:** 1 Laboratory of Signal Transduction, Nencki Institute of Experimental Biology, Warsaw, Poland; 2 Department of Medicine, Monash University Central Clinical School, Prahran, Victoria, Australia; 3 Alfred Hospital, Prahran, Victoria, Australia; Ohio State University Medical Center, United States of America

## Abstract

The Grainyhead-like 1 (GRHL1) transcription factor regulates the expression of desmosomal cadherin desmoglein 1 (*Dsg1*) in suprabasal layers of the epidermis. As a consequence, the epidermis of *Grhl1*-null mice displays fewer desmosomes that are abnormal in structure. These mice also exhibit mild chronic skin barrier defects as evidenced by altered keratinocyte terminal differentiation, increased expression of inflammatory markers and infiltration of the skin by immune cells. Exposure of *Grhl1* ^−/−^ mice to a standard chemical skin carcinogenesis protocol results in development of fewer papillomas than in wild type control animals, but with a rate of conversion to squamous cell carcinoma (SCC) that is strikingly higher than in normal littermates. The underlying molecular mechanism differs from mice with conditional ablation of a closely related Grhl family member, *Grhl3*, in the skin, which develop SCC due to the loss of expression of phosphatase and tensin homolog (PTEN) and activation of the phosphatidylinositol 3-kinase (PI3K)/AKT/mechanistic target of rapamycin (mTOR) signaling pathway.

## Introduction

Skin, a critical organ in terrestrial animals, constitutes the boundary between organism’s interior and external environment, limiting water loss and protecting from mechanical, chemical and pathogenic insults. The functional barrier is formed by the epidermis – the most external layer of the skin. Keratinocytes, cells that make up the epidermis, undergo terminal differentiation – specific and tightly regulated form of cell death. During this process the remains of cells are cross-linked with proteins and lipids, forming hydrophobic and mechanically resistant layer. Proper barrier formation depends on cell-cell junctions, which include desmosomes, adherens junctions and tight junctions, and disruption of their protein content or architecture can cause abnormalities in skin function, eventually leading to diseases ranging from atopic skin reactions to more severe disorders such as palmoplantar keratoderma.

Desmosomes are composed of transmembrane cadherin family members – desmogleins (DSGs) and desmocollins (DSCs), as well as the proteins from plakin family (desmoplakin – DSP) and armadillo family (plakoglobin – JUP and plakophilins – PKPs). Cadherins form homo- and heterodimers with proteins on adjacent cells. Such complexes are connected via JUP, PKPs and DSP with cellular cytoskeleton – intermediate filaments, mainly from the keratin family. Because of their association with keratins, for many years desmosomes were assumed to be passive junctions, responsible only for mechanical endurance of the epidermis. Research of the last 10 years revealed their functions as “connectivity receptors”. Today they are recognized as active players in cellular signaling pathways, many of which are linked to cancer development [1]. For instance, DSG1 suppresses the epidermal growth factor receptor (EGFR)/mitogen-activated protein kinase (MAPK) pathway during epidermal differentiation [2], DSC3 functions as a tumor suppressor by inhibiting the EGFR/extracellular signal-regulated kinase (ERK) signaling in human lung cancer [3], DSP acts as a tumor suppressor by inhibiting the wingless-type mouse mammary tumor virus integration site family (Wnt) signaling pathway in human lung cancer [4] and JUP indirectly inhibits Rous sarcoma oncogene (Src) kinase in prostate cancer [5]. Little is known about the involvement of desmosomal components in the development of skin cancers. The most likely candidates for suppressors or supporters of this type of malignancies are proteins responsible for the proper development of epidermis and formation of epidermal barrier.

The transcription factors from the Grainyhead-like (GRHL) family are highly expressed in the epidermis and are crucial for the accurate development and functional features of this organ, even in such evolutionarily distant organisms as insects and mammals. This family has three mammalian members, which are termed GRHL1-3 [6], [7]. Previously we reported links between GRHL3 and skin cancer [8]. Mice with skin-specific ablation of *Grhl3* display increased propensity to chemically-induced skin tumorigenesis, and the expression of *GRHL3* is significantly reduced in human SCC samples, compared to the adjacent epidermis. The molecular mechanism underlying the role of GRHL3 in skin cancer is dependent on direct regulation of expression of tumor suppressor PTEN by GRHL3. In human skin tumors both *GRHL3* and *PTEN* are regulated by micro RNA miR-21 [8].

The role of GRHL1 in skin carcinogenesis has not been investigated before. Previously we reported that this transcription factor directly regulates the expression of *Dsg1* and in the epidermis of *Grhl1* ^−/−^ mice the desmosomes are fewer in numbers and have abnormal structure [9]. Consequently, epidermis of these animals is thicker and we observe perturbed expression of differentiation markers. On this basis we proposed a hypothesis that the *Grhl1*-null mice have altered susceptibility to the standard chemical skin carcinogenesis protocol. In this article we would like to present results concerning the role of GRHL1 transcription factor in maintenance of the epidermis, in the immunology of the skin, and in skin cancer development.

## Materials and Methods

### Ethics Statement

This study was carried out in strict accordance with the regulations of the Experiments on Animals Act (Act of 21 January 2005 on experiments on live animals, the Parliament of the Republic of Poland, Dz. U. Nr 33, poz. 289); as well as with the Directive 2010/63/EU of the European Parliament and of the Council of the European Union of 22 September 2010 on the protection of animals used for scientific purposes. All animal experiments were approved by the First Warsaw Local Ethics Committee for Animal Experimentation; permit number 1042/2009. All efforts were made to minimize suffering.

### Skin Microscopic Sample Preparation

Mice of age about 6 months were sacrificed and shaved back skin sections were dissected, fixed in 4% paraformaldehyde (Acros Chemicals, Geel, Belgium) in phosphate-buffered saline (PBS) and embedded in paraffin (POCH, Gliwice, Poland). Samples were cut into 10 or 7 µm sections using microtome Hyrax M55 (Zeiss, Jena, Germany) and placed on Superfrost Ultra Plus microscope slides (Thermo Scientific, Waltham, MA, USA). Sections were then deparaffinized with xylene and decreasing concentrations of alcohols.

### Immunohistochemistry and Immunofluorescence

Prepared 7 µm skin sections were incubated in citrate buffer at 60°C overnight (antigen retrieval). The endogenous peroxidase activity was blocked by incubation in 1% hydrogen peroxide in PBS for 15 minutes. The following rabbit polyclonal anti-mouse antibodies were used: anti-involucrin (PRB-140C), anti-filaggrin (PRB-417P), anti-loricrin (PRB-145P), anti-keratin 5 (PRB-160P), anti-keratin 6 (PRB-169P), anti-keratin 10 (PRB-159P) (all from Covance, Princeton, NJ, USA). The rabbit monoclonal anti-Ki67 antibody (ab16667) was purchased from Abcam (Cambridge, UK). For immunodetection the 3,3′-diaminobenzidine (DAB) Detection Kit (USA™ Ultra Streptavidin Detection System, Covance, SIG-32232) was used according to the manufacturer’s instructions. For immunofluorescence, we followed the Immunofluorescence Standard Protocol published by the Cell Signaling Technology company. The results were documented using microscope (Eclipse 80i, Nikon, Tokyo, Japan) with digital camera.

### Skin Infiltration by Mast Cells

Procedure as described in [10]. Skin samples from four *Grhl1* ^−/−^ and five *Grhl1* ^+/+^ mice were collected. Four different samples from every animal were paraffinized and cut to 10 µm sections. After deparaffinization they were incubated in acidic toluidine blue (Sigma-Aldrich, Munich, Germany) solution (0.1% toluidine blue, 7% ethanol, 1% sodium chloride, pH 2.3) for 5 minutes, thoroughly washed in water and dehydrated in increasing concentrations of alcohols, xylene and mounting medium (DePeX). Using microscope (Eclipse 80i, Nikon) purple mast cells between epidermis and dermal muscle layer were counted. Areas of interest were assessed using ImageJ software.

### RNA and cDNA Preparation, Quantitative Real Time Polymerase Chain Reaction (Q-RT-PCR)

Mice of age about 6 months were sacrificed, their back skin dissected and immediately frozen in liquid nitrogen. The samples were ground in mortar in liquid nitrogen, the Ron’s FastTRI Extraction Reagent (Bioron, Ludwigshafen, Germany) was added and the solution was homogenized using Polytron (PRO2000, PRO Scientific, Oxford, CT, USA). The RNA was isolated according to the manufacturer’s instructions. The RNA was then reverse transcribed into cDNA using Moloney Murine Leukemia Virus (M-MuLV) reverse transcriptase (Bioron) according to the producer’s protocol. The Q-RT-PCR reactions were carried out using SYBR Green PCR Mastermix on 7500 Real Time PCR System (Applied BioSystems, Grand Island, NY, USA). Used primers are listed in Table S3. Relative expression levels were standardized to hypoxanthine phosphoribosyltransferase (HPRT) expression, and statistical differences were determined by Student’s t-test. In the assay of S100A8 expression, we used six *Grhl1* ^−/−^ and six *Grhl1* ^+/+^ animals; in the assay of S100A9 expression – five *Grhl1* ^−/−^ and five *Grhl1* ^+/+^ mice.

### Thymic Stromal Lymphopoietin (TSLP) Level Assay

The blood level of TSLP was measured using enzyme-linked immunosorbent assay (ELISA) kit: Quantikine Mouse TSLP (R&D Systems, Minneapolis, MN, USA) according to the manufacturer’s instructions, on groups of seven *Grhl1* ^−/−^ and five *Grhl1* ^+/+^ animals, at the age of about 4 months. Statistical differences were determined by Student’s t-test.

### Skin Carcinogenesis Protocol

To determine the role of GRHL1 transcription factor in skin carcinogenesis we utilized the well established protocol of chemically induced skin cancers [11]. Briefly, animals at 8–12 weeks of age (n = 15 per group) were shaved and topically treated with single application of tumor initiator 7,12-dimethylbenz[α]anthracene (DMBA) (25 µg dissolved in 150 µl of acetone) followed by twice weekly application of tumor promoter phorbol 12-tetradecanoate 13-acetate (TPA) (7.6 nanomoles dissolved in 200 µl of acetone). The treatment lasted for 30 weeks. Developing papillomas were counted when they reached at least 1 mm in diameter, their appearances were different from the adjacent tissue and remained visible for at least two weeks. Squamous cell carcinomas were counted as lesions that were at least 3.5 mm in diameter and acquired a round, crateriform shape. For ethical reasons mice which developed tumors larger than 1 cm in diameter were sacrificed. Measurements were performed using ImageJ software. Representative groups of papillomas and carcinomas were histologically analyzed by a certified pathologist – Monika Durzynska, MD, from the Maria Sklodowska-Curie Memorial Cancer Center and Institute of Oncology in Warsaw.

### Statistical Analysis

The statistical analysis was performed using Student’s t-test incorporated into Microsoft Office Excel 2003 package.

## Results

### Keratinocytes in *Grhl1* ^−/−^ Epidermis Undergo Severely Deregulated Terminal Differentiation

**Figure 1:**
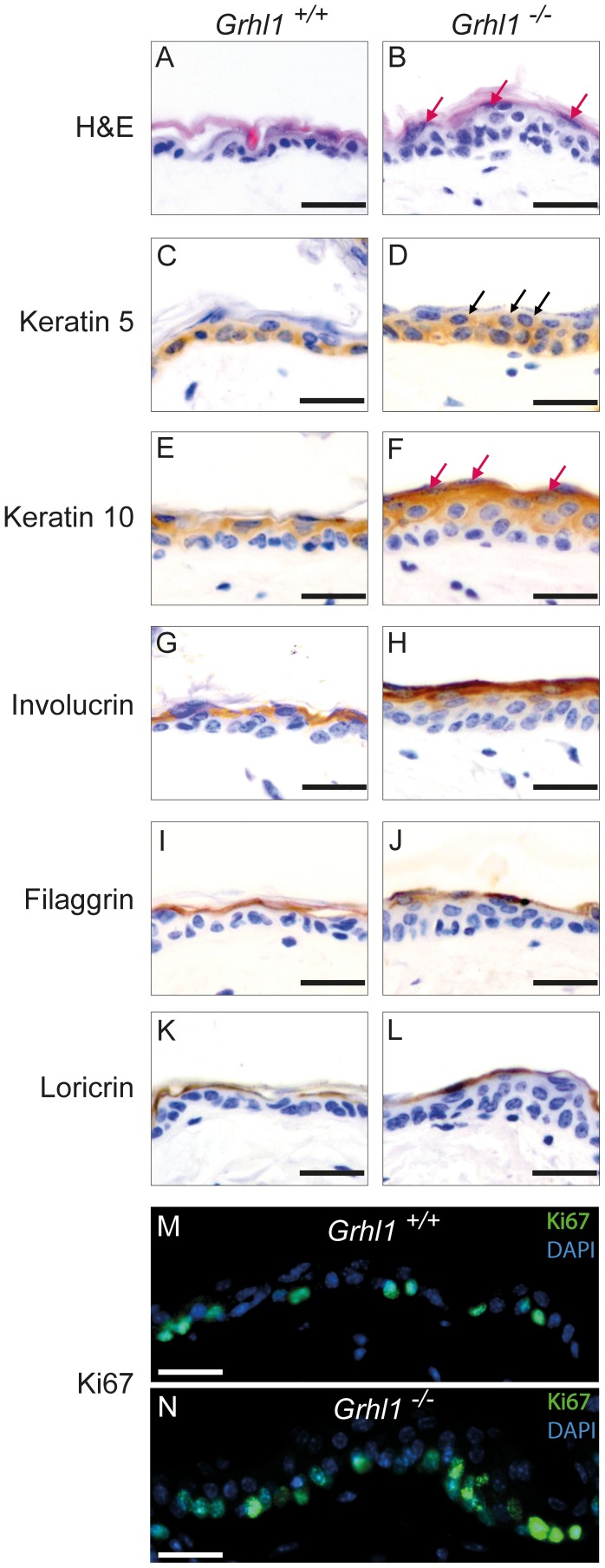
Histological analysis of the skin of *Grhl1* ^−/−^ mice (right panels) in comparison to wild type littermates *Grhl1* ^+/+^ (left panels). Scale bars represent 25 µm. (A–B) Hematoxylin and eosin (H&E) staining of epidermis of *Grhl1* ^+/+^ and *Grhl1* ^−/−^ mice; red arrows point to keratinocytes from granular layer of epidermis. (C–L) Immunohistochemical analysis of markers of epidermal differentiation in the epidermis of *Grhl1* ^+/+^ and *Grhl1* ^−/−^ mice: marker of basal layer – keratin 5 (C, D, black arrows indicate suprabasal keratinocytes expressing keratin 5), marker of early terminal differentiation – keratin 10 (E, F, red arrows point to keratinocytes from the granular layer of epidermis), markers of late terminal differentiation – involucrin (G, H), filaggrin (I, J), loricrin (K, L). (M, N) Immunofluorescence staining of proliferation marker Ki67 in *Grhl1* ^+/+^ (M) and *Grhl1* ^−/−^ (N) mice.

To further characterize the functions of GRHL1 transcription factor, we carried out a detailed examination of the skin of *Grhl1-*null mice. In our previous work we investigated the expression of various keratins and involucrin only on the palmoplantar surfaces of the paws [9]. Now we focused our attention on the expression of relevant markers in the back skin. By histological analysis we showed that epidermis from the back skin of *Grhl1* ^−/−^ mice is thicker than in wild type littermates (Fig. 1A–B). Increased thickness of epidermis is recognized as one of the indicators of epidermal defects. To create functional barrier layer of the epidermis, which is formed between stratum granulosum and stratum corneum, it is crucial for epidermal keratinocytes to undergo proper terminal differentiation. To investigate this process in *Grhl1* ^−/−^ mice we performed immunohistochemical detection of specific markers (Fig. 1C–L). Epidermal cells of knockout animals undergo full program of terminal differentiation. Keratinocytes of basal layer (with additional cells in suprabasal layers, see later) express basal marker keratin 5, in suprabasal layers there is expression of early (keratin 10) and late differentiation markers (involucrin, filaggrin and loricrin). Cells with characteristic dense cytoplasmic granules, characteristic of stratum granulosum can be easily distinguished (Fig. 1B, F, red arrows). What is noteworthy, cells positive for keratin 5 (marker of basal keratinocytes) can also be detected in suprabasal layers of epidermis (Fig. 1D, black arrows). Furthermore, the expression of a proliferation marker Ki67 is increased in the *Grhl1* ^−/−^ epidermis (Fig. 1M–N). This suggests that the suprabasal keratinocytes in the skin of *Grhl1-*null mice exhibit severe deregulation of terminal differentiation program, which may lead to thickening of the epidermis and subacute impairment of epidermal barrier.

### *Grhl1* ^−/−^ Mice Exhibit Mild Chronic Skin Barrier Defects

**Figure 2:**
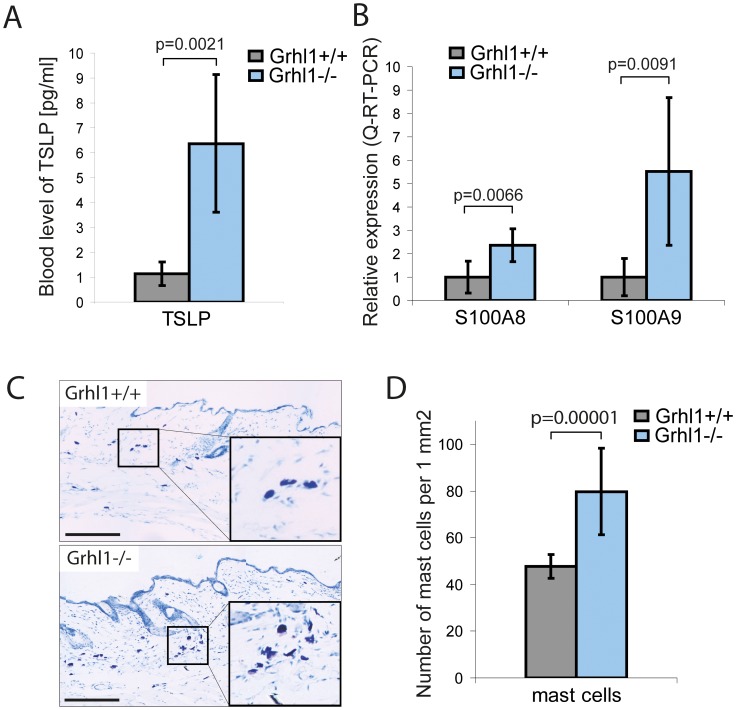
Skin inflammation in *Grhl1* ^−/−^ mice. (A) Blood levels of TSLP in *Grhl1* ^+/+^ (gray bar) and *Grhl1* ^−/−^ mice (blue bar), measured with ELISA kit. (B) Levels of expression of antimicrobial peptides S100A8 and S100A9 in the epidermis of *Grhl1* ^+/+^ (gray bar) and *Grhl1* ^−/−^ mice (blue bar), measured with Q-RT-PCR. (C) Representative skin sections of *Grhl1* ^+/+^ (top panel) and *Grhl1* ^−/−^ mice (bottom panel) stained with toluidine blue to visualize dermal mast cells (purple cells). Scale bars represent 200 µm. (D) Quantification of skin infiltration with mast cells for *Grhl1* ^+/+^ (gray bar) and *Grhl1* ^−/−^ mice (blue bar), estimated as numbers of stained cells per 1 mm^2^ area of 10 µm thick skin section (using ImageJ software). (A, B, D) Significance (Student’s t-test, p-value) is shown above bars.

Mild impairments in epidermal barrier function are often accompanied by chronic activation of skin’s immune system, hence specific markers of inflammation can serve as indicators of barrier function [12]. For that reason we investigated the activation of skin’s immune system in *Grhl1* ^−/−^ mice. One of the markers of disrupted epidermal barrier and skin’s allergic reaction is cytokine TSLP. Its high expression in keratinocytes and elevated level in blood is correlated with epidermal dysfunction and chronic inflammation [13]. Using ELISA we measured the level of this cytokine in the blood of *Grhl1* ^−/−^ mice and their wild type littermates. In the blood of knockout animals we detected significantly higher levels of TSLP than in the wild type controls (Fig. 2A; p = 0.0021). We employed the Q-RT-PCR method to measure the expression of other markers of subacute disruption of skin barrier – antimicrobial peptides S100A8 and S100A9 [14]. We detected significantly increased levels of expression of both these markers in knockout animals’ epidermis. Relative expression of S100A8 in *Grhl1* ^−/−^ mice was 2.37, SD = 0.71; and in the wild type controls 1.00, SD = 0.68 (p = 0.0066). In the case of S100A9, relative expression in *Grhl1* ^−/−^ mice was 5.52, SD = 3.15, and in the wild type controls 1.00, SD = 0.79 (p = 0.0091) (Fig. 2B).

Antimicrobial peptides and TSLP are known to act as chemo-attractants and their elevated levels in the skin induce its infiltration with immune cells, as detailed in a recent review [15]. To detect immune cells in the dermis we stained skin sections with toluidine blue, which labels cells containing granules rich in histamine and heparin, mainly mast cells. After such staining they are visible as purple cells. Data was documented by photography (Fig. 2C). Results were quantified using ImageJ software as a number of cells per 1 mm^2^ of 10 µm thick section of skin. *Grhl1* ^−/−^ mice have significantly more stained cells (79.8 cells/mm^2^; SD = 18.5 cells/mm^2^) than their wild type littermates (47.7 cells/mm^2^; SD = 5.0 cells/mm^2^) (p = 0.00001) (Fig. 2D). These results indicate that the *Grhl1* ^−/−^ mice exhibit mild chronic skin inflammation.

### Grhl1−/− Mice Develop more Skin Tumors with Earlier Onset than Grhl1+/+ Littermates

**Figure 3:**
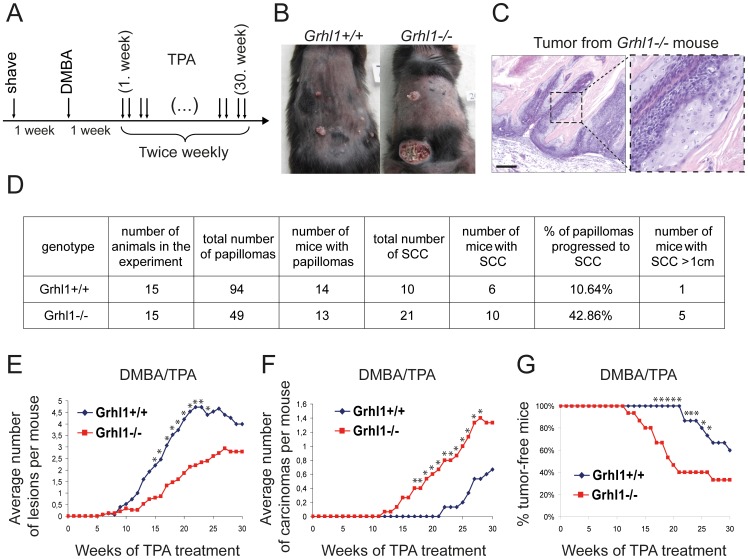
Skin carcinogenesis in *Grhl1* ^−/−^ and control mice. (A) Timeline of DMBA/TPA treatment in two-stage chemically-induced skin tumor development protocol. (B) Representative *Grhl1* ^−/−^ and *Grhl1* ^+/+^ mice after 30 weeks of TPA treatment. (C) Hematoxylin and eosin staining of a representative SCC tumor from a *Grhl1* ^−/−^ mouse at low (left panel) and high magnification (right panel); scale bar represents 200 µm. (D) Summary of the results. (E–F) Average number of: (E) papillomas; (F) squamous cell tumors; (G) Kaplan-Meier analysis presenting tumor free mice in relation to the time of experiment. *Grhl1* ^−/−^ – red lines; *Grhl1* ^+/+^ – blue lines. Asterisks indicate time points at which differences between the two groups of animals were statistically significant (p<0.05).

It has recently been proposed that any mouse model with subacute skin barrier defects may display increased propensity to chemically-induced skin tumor development [16]. *Grhl1* ^−/−^ animals do not develop spontaneous tumors, even in old age, and the observable lifespan and activity of these mice in old age are the same as in control wild type littermates (Table S1). This suggests that loss of GRHL1 transcription factor alone is insufficient to induce cancerous transformation. However, the genetic background of *Grhl1* KO mice is C57BL/6 (Black 6), and this mouse strain is very resistant to skin tumorigenesis, which may explain why we did not observe spontaneous tumors in the *Grhl1*-null mice [17]. Therefore we applied the standard two-stage skin carcinogenesis protocol in *Grhl1* ^−/−^ and *Grhl1* ^+/+^ mice, using DMBA and TPA as tumor inducer and promoter, respectively (Fig. 3A) [11], [18]. In this experiment we used 15 mice of each genotype, at the starting age of 2–3 months. The representative appearance of *Grhl1* ^−/−^ and *Grhl1* ^+/+^ mice after 30 weeks of TPA treatment is presented in Fig. 3B. The histological analysis was performed by certified pathologist – Monika Durzynska, MD, from the Maria Sklodowska-Curie Memorial Cancer Center and Institute of Oncology, Warsaw. This assay revealed no differences in types of lesions between control and *Grhl1* ^−/−^ mice, and all analyzed samples were papillomas or highly differentiated SCC. In the wild type mice, 26 overall lesions (28%) were examined by the certified pathologist, which included 10 SCC (100%). In the *Grhl1*-null mice, 32 overall lesions (65%) were examined by Ms Durzynska, which included 12 SCC (57%). The hematoxylin and eosin staining of a representative squamous cell tumor from a knockout animal is presented on Fig. 3C.

We did not observe significant differences between *Grhl1* ^+/+^ and *Grhl1* ^−/−^ mice in the onset of development of papillomas. In both groups the first papillomas appeared after 5 weeks of TPA treatment (Fig. 3E). In total the *Grhl1* ^−/−^ mice developed 49 papillomas, *Grhl1* ^+/+^ –94 papillomas (p = 0.022). The number of mice which developed papillomas was similar –14 and 13 mice, respectively, out of 15 mice in each group (Fig. 3D). These results suggest again that loss of GRHL1 transcription factor has no impact on timing of cancerous transformation of keratinocytes, but it reduces the occurrence of papillomas.

During the experiment some papillomas regressed. This affected 32 papillomas (34%) in *Grhl1* ^+/+^ mice and 4 papillomas (8%) in *Grhl1* ^−/−^ mice. Fig. 3D provides the sum total of all the papillomas that appeared on all the mice of a particular genotype during the experiment, and diagram 3E shows the average number of papillomas per mouse at given time points. Some papillomas progressed to SCC (Fig. 3D). In total *Grhl1* ^−/−^ mice developed 21 such tumors and wild type littermates –10; SCC arose in 10 *Grhl1*-null and 6 wild type mice. What is noteworthy, in *Grhl1* ^−/−^ mice almost 43% of papillomas progressed to SCC, whereas in the control animals – fewer than 11% (p = 0.0031). The onset of SCC was also accelerated – the first carcinomas in *Grhl1*-null mice were observed after 12 weeks of TPA treatment, and in *Grhl1* ^+/+^ mice – after 22 weeks (p = 0.0007) (Fig. 3F–G). Moreover, we also observed differences in size of carcinomas – in the case of *Grhl1* ^+/+^ mice only one animal developed SCC larger than 1 cm in diameter before the 30^th^ week of experiment and had to be sacrificed for ethical reasons, whereas in *Grhl1* ^−/−^ mice – five animals had to be sacrificed for these reasons (Fig. 3D). This suggests that loss of *Grhl1* accelerates progression from benign papilloma to SCC, and positively influences the size of carcinomas.

## Discussion

Our research interests concern the GRHL1 transcription factor and its role in the skin. Previously we demonstrated that this protein is confined to differentiating subrabasal keratinocytes in the epidermis and to the inner root sheath of hair follicle, but is absent from the dermal papilla [9]. The *Grhl1* ^−/−^ mice are viable and fertile, but they show initial delay in coat growth, and older mice have sparse fur and poor anchorage of hair shaft in the follicle which leads to extensive hair loss. They also display thickening of the epidermis on the palmoplantar surfaces of their paws, which is reminiscent of palmoplantar keratoderma, a disorder caused by mutations in the *DSG1* gene in human patients [19], [20]. Accordingly, the epidermal desmosomes in *Grhl1*-null mice are shorter, less well organized and sensitive to ethylene glycol tetraacetic acid (EGTA), which is indicative of their reduced stability [9].

Here we present our results of a detailed analysis of epidermal function in the *Grhl1* ^−/−^ mice. Previously we reported that the GRHL1 transcription factor regulates the expression of a gene coding for desmosomal cadherin desmoglein 1 (*Dsg1*) [9]. This protein is a main constituent of cell-cell adhesion complexes between suprabasal keratinocytes – desmosomes, and is also a regulator of induction of terminal differentiation of keratinocytes [2]. What is noteworthy, we have shown before that the levels of expression of other markers of basal keratinocytes – DSG2 and DSG3– are increased in *Grhl1*-null mice [9]. These animals display the thickening of squamous layer. Thickening of the epidermis over areas exposed to mechanical forces is a typical response of healthy skin, but it may occur at low levels of mechanical stress if the mechanical endurance of the skin is compromised [21]. In the *Grhl1* ^−/−^ mice the level of DSG1 is insufficient for formation of properly composed suprabasal desmosomes, which results in numerous desmosomal defects and is likely to reduce the mechanical resistance of the skin. The observed epidermal response was exclusively dependent on changes in DSG1 expression, as the levels of components of other cell adhesion complexes are not altered in the *Grhl1* ^−/−^ mice [9]. GRHL1 is expressed in the same epidermal cells as DSG1, and in the *Grhl1* ^−/−^ animals the production of this cadherin is reduced, which leads to the thickening of innermost, keratin 5-positive cell layer. By study of other differentiation markers we demonstrated that the suprabasal keratinocytes in the skin of *Grhl1-*null mice exhibit severe deregulation of terminal differentiation program, which may lead to thickening of the epidermis and subacute impairment of epidermal barrier.

In our subsequent experiments we investigated whether the *Grhl1* ^−/−^ mice display symptoms of skin barrier defects. In the mutant animals we observed elevated blood levels of a marker of response to barrier defects TSLP, an interleukin-7-like cytokine. When secreted by epithelial cells, this factor stimulates tissue infiltration with immune cells and facilitates their activation [13]. Other markers of defective barrier are antimicrobial peptides S100A8 and S100A9. Genes coding for these Ca^2+^-dependent alarm factors belong to epidermal differentiation complex (EDC) [22], [23]. In the unaffected epidermis the expression of S100A8 and S100A9 is low, but it is strongly stimulated upon induction of chronic inflammation. These proteins act as chemoattractants and their elevated secretion by keratinocytes induces dermal infiltration by immune cells. In addition, S100 proteins are increasingly recognized as major regulators of tumor promoting inflammatory microenvironment [24]. Therefore we measured the levels of these markers in the epidermis of *Grhl1* ^−/−^ mice and we observed increased expression of S100A8 and S100A9. Moreover, the expressional microarray on whole skin (epidermis and dermis) of *Grhl1* ^−/−^ mice (Table S2 and [9]) revealed upregulation of genes from the small proline-rich family (SPRR) which also belong to EDC – *Sprr2d* (fold change more than 2), as well as *Sprr2g*, *Sprr2e*, *Sprr2a*, *Sprr1a* (fold change between 1 and 2). SPRR proteins form scaffold for other proteins in the cornified envelope of epidermis and are highly upregulated in the skin of barrier-deficient mouse models [22], [25]. Furthermore, the upregulation of other genes associated with inflammation was also detected in this experiment, including the chemokines from chemokine C-X-C motif ligand (CXCL) family *Cxcl1*, *Cxcl9*, *Cxcl16*; beta-defensins *Defb4*, *Defb6*, *Defb14*; and others. Many of them have been associated with cancerous transformation (Table 1). Among the upregulated genes we also discovered genes coding for proteins specific for mast cells – multiple C2 domains, transmembrane 1 (*Mctp1*), *Mctp4* and protease, serine, 22 (*Prss22*) (Table S2). This prompted us to assess the number of immune cells infiltrating the skin. In the skin from *Grhl1* ^−/−^ mice we detected increased count of toluidine blue stained cells, that are recognized as mast cells. These are known to support the development of SCC [26]. The described gene signature and infiltration with immune cells agree with the notion that the *Grhl1* ^−/−^ mice exhibit mild skin barrier impairments.

**Table 1:** List of some of the genes that are upregulated in the *Grhl1* ^−/−^ skin.

Gene name	Accession no.	Literature links to inflammation	Literature links to cancer
Cxcl1	NM_008176.1	[38]	[39], [40]
Cxcl9	NM_008599.1	[38]	[41], [42]
Cxcl16	NM_023158.3	[38], [43]	[44], [45]
Defb4	NM_019728.2	[46], [47]	
Defb6	NM_054074.1	[48]	
Defb14/Defb3	NM_183026.1	[47]	[49]
Prss18/Klk6	NM_011177	[50], [51]	[52], [53]
Prss22	NM_133731.1	[54]	
Slpi	NM_011414.1	[55], [56]	[57]
Stfa1	NM_001001332	[58]	[59], [60]
Sprr2d	NM_011470.1	[61]	
Saa1	NM_009117.1	[62]	[63], [64]
Saa3	NM_011315	[65]	[65]
Hpxn	NM_017371.1	[66]	
Hp	NM_017370.1	[66]	
Aldh3a1	NM_007436.1	[67]	
Serpina1b	NM_009244.2	[68]	[69]
Ier3	NM_133662.1	[70]	[70]

Full list of genes with fold changes higher than 2 is provided in Table S2.

Recent research showed that mice with subacute skin barrier defects may be more prone to chemically-induced skin tumor development [16]. The authors suggested that the underlying mechanism is general and is dependent on development of tumor-promoting chronic inflammatory microenvironment in the skin. Here we present data concerning the role of GRHL1 transcription factor in development of skin cancers. The *Grhl1*-null mice do not develop spontaneous tumors, even in old age, and their life span is the same as that of their *Grhl1* ^+/+^ littermates (Table S1). In the standard skin chemical carcinogenesis protocol the timing of papilloma development is the same in the *Grhl1* ^−/−^ mice and in their wild type littermates. This observation suggests that loss of *Grhl1* has no impact on timing of initiation of cancerous transformation of keratinocytes. However, the mutant mice developed significantly fewer papillomas, but the progression to squamous cell tumors was dramatically increased, with almost 43% of papillomas progressing to carcinomas, in comparison with wild type littermates, where the progression rate was under 11%. The tumors in knockout mice were also bigger in size and had earlier onset. In the light of these results we can state that loss of *Grhl1* supports the progression of papillomas to carcinomas in a mouse model.

The *Grhl1* ^−/−^ mice develop more carcinomas but fewer papillomas than their wild type littermate controls. This is a very unusual phenotype, but it is reminiscent of the *p53*-deficient mice [27]. p53 is a well known tumor suppressor [28]. Surprisingly, following the DMBA/TPA chemical skin carcinogenesis protocol, the *p53* ^−/−^ mice developed about five times fewer papillomas than their *p53* ^+/+^ and *p53* ^+/−^ littermates, and this result was statistically significant. In contrast, the frequency of conversion from papillomas to carcinomas was increased from 3% in wild type mice to 43% in *p53*-null mice. The onset of papillomas was the same in *p53* ^−/−^, *p53* ^+/−^ and *p53* ^+/+^ animals, but the onset of SCC in *p53*-null mice was much earlier than in their wild type and heterozygous littermates. The authors also reported regression of some papillomas in the *p53* ^+/+^ and *p53* ^+/−^ mice during the last five weeks of the experiment [27]. We obtained very similar results for the *Grhl1* knockout mice (Fig. 3D–G). The increased conversion rate from papillomas to SCC in the *p53*-null mice can be explained by the tumor suppressor properties of p53, while the decreased occurrence of papillomas in *p53* ^−/−^ mice is most likely caused by the activation of p53-independent apoptotic pathway [29]. It is thus possible that analogous mechanisms are responsible for the involvement of GRHL1 in skin carcinogenesis.

Yet another possible explanation for the decrease in the number of papillomas accompanied by an increased number of carcinomas is provided by recent research proposing different cells of origin of long-lived papillomas and papillomas progressing to carcinomas [30]–[33]. The former originate from the interfollicular epidermis, while the latter – from keratinocytes of the hair follicle. We have reported before that the epidermal defects in *Grhl1* ^−/−^ mice are relatively minor: the impermeability of skin barrier is not compromised; the Nikolsky sign for epidermal fragility is negative; and wound healing is unaffected. On the other hand, the impairments of hair follicles are much more severe: the hair anchoring is defective, and the structure of depilated hair follicles is completely different from the controls [9]. Thus the desmosomal defects in the *Grhl1* ^−/−^ mice are likely to have different effects on the keratinocytes in hair follicles and in interfollicular epidermis, which could explain why the mutant mice develop fewer papillomas but more carcinomas in skin carcinogenesis experiments.

There are several other mechanisms that may contribute towards the observed phenotype of *Grhl1* ^−/−^ mice, and these will need to be investigated in future studies. There may exist as yet unidentified additional target genes of GRHL1 regulation whose expression is dysregulated in the *Grhl1*-null mice, and this dysregulation may play a part in the decrease in papilloma incidence and increased conversion rate of papillomas to SCC. Another plausible mechanism involves changes to stem cells: the interfollicular stem cells may be impaired, while the hair follicle bulge stem cells may be expanded. The balance between proliferation and apoptosis may be disrupted in the *Grhl1* ^−/−^ epidermis as well. All these alternative mechanisms may be relevant to skin carcinogenesis in the *Grhl1*-null mice.

In our previous publication we described links between the GRHL3 transcription factor and skin cancer [8]. In that paper we reported that similarly to GRHL1, GRHL3 also has a protective role against skin carcinogenesis. Here we demonstrate that, even though GRHL1 and GRHL3 share a high degree of sequence homology, have almost identical target DNA binding sites and display very similar expression patterns in the epidermis [34], the molecular mechanisms linking them to cancer are very different. GRHL3 inhibits epidermal tumorigenesis through direct regulation of tumor suppressor PTEN. Here we present evidence that GRHL1 does not regulate the expression of PTEN (Fig. S1), and that it protects against skin carcinogenesis by regulating the maintenance of skin barrier. This observation is consistent with our earlier report that, despite recognizing the same consensus DNA binding sequence, in living cells different GRHL factors regulate the expression of different target genes [35].

In summary, we report here that the *Grhl1*-null mice display increased susceptibility to chemically induced SCC. The underlying molecular mechanism is most likely related to the subacute skin barrier defects in these mice. Similar observations have been made using other mouse models; however, the molecular pathway has never been delineated that would link skin barrier impairments to increased tumor occurrence. At the present moment it is known that regardless of the cause of skin barrier disruption; whether it is brought about by alterations in the Notch signaling pathway [16], or in the Wnt signaling pathway [36], or by targeting a desmosomal component [37], or by ablation of the *Grhl1* gene (present study); in all these cases a not yet characterized skin barrier-dependent, tumor-promoting pathway is triggered which leads to increased cancer susceptibility. Characterizing this pathway presents a very important direction for future research.

## Supporting Information

Figure S1 **Relative expression of** ***Pten*** **gene in the epidermis of** ***Grhl1*** **-null mice and control animals, measured by Q-RT-PCR (p = 0.96).**
(TIF)

Table S1 **Age-related spontaneous cancer development in** ***Grhl1*** **-null mice and control animals.**
(DOC)

Table S2 **Upregulated genes (fold change min. 2.0) in** ***Grhl1*** **^−/−^ mouse skin.**
(DOC)

Table S3 **List of primers used in Q-RT-PCR. The abbreviations are: R – reverse, F – forward.**
(DOC)

Materials and Methods S1 **Spontaneous cancers development.** Microarray analysis of *Grhl1* ^+/+^ and *Grhl1* ^−/−^ mouse skin.
(DOC)
