# Supplementary figures and images for: Loss of Grainy Head-Like 1 Is Associated with Disruption of the Epidermal Barrier and Squamous Cell Carcinoma of the Skin
Source: PLoS One. 2014 Feb 20;9(2):e89247. doi: 10.1371/journal.pone.0089247 (PMC3930704; doi:10.1371/journal.pone.0089247)

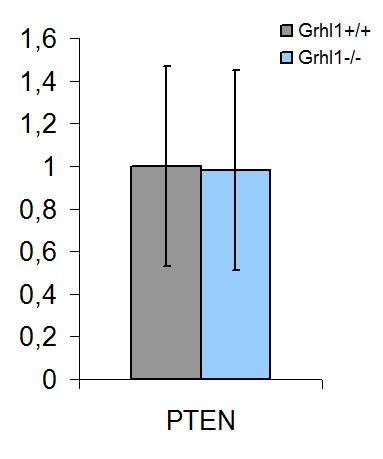

Supplement: Figure S1 — Relative expression of Pten gene in the epidermis of Grhl1 -null mice and control animals, measured by Q-RT-PCR (p = 0.96). (TIF) [file pone.0089247.s001.tif]
