# Supplementary material for: Loss of Grainy Head-Like 1 Is Associated with Disruption of the Epidermal Barrier and Squamous Cell Carcinoma of the Skin
Source: PLoS One. 2014 Feb 20;9(2):e89247. doi: 10.1371/journal.pone.0089247 (PMC3930704; doi:10.1371/journal.pone.0089247)
Supplement: Table S1 — Age-related spontaneous cancer development in Grhl1 -null mice and control animals. (DOC) [file pone.0089247.s002.doc]

**Table S1.** Age-related spontaneous cancer development in *Grhl1*-null mice and control animals.

|  | number of mice in experiment | number of mice that died before the age of 30 months | number of mice with visible skin tumors | number of mice with other visible tumors or abnormalities |
| --- | --- | --- | --- | --- |
| *Grhl1*^+/+^ | 13 | 1 | 0 | 0 |
| *Grhl1*^-/-^ | 13 | 2 | 0 | 1* |

* unrelated dysfunction
