# Supplementary material for: Loss of Grainy Head-Like 1 Is Associated with Disruption of the Epidermal Barrier and Squamous Cell Carcinoma of the Skin
Source: PLoS One. 2014 Feb 20;9(2):e89247. doi: 10.1371/journal.pone.0089247 (PMC3930704; doi:10.1371/journal.pone.0089247)
Supplement: Table S2 — Upregulated genes (fold change min. 2.0) in Grhl1 −/− mouse skin. (DOC) [file pone.0089247.s003.doc]

**Table S2.** Upregulated genes (fold change min. 2.0) in *Grhl1*^-/-^ mouse skin.

| microarray hit | accession | fold change | p |
| --- | --- | --- | --- |
| Saa3 | NM_011315 | 22.66 | 1.55E-12 |
| Stfa1 | NM_001001332 | 15.00 | 4.62E-11 |
| Lcn2 | NM_008491.1 | 9.77 | 2.89E-12 |
| LOC268393 | XM_196027.3 | 9.09 | 2.52E-08 |
| Prss18 | NM_011177 | 8.56 | 2.75E-10 |
| AA467197 | NM_001004174.1 | 7.85 | 9.44E-12 |
| Hpxn | NM_017371.1 | 7.72 | 8.77E-11 |
| Chi3l1 | NM_007695.1 | 7.53 | 1.24E-09 |
| C920013G19Rik | AK050607 | 6.20 | 3.54E-07 |
| Slpi | NM_011414.1 | 5.21 | 3.91E-10 |
| Defb14 | NM_183026.1 | 4.47 | 3.77E-11 |
| 4732456N10Rik | NM_177717.2 | 4.19 | 2.05E-07 |
| 4833405L16Rik | NM_177197.2 | 4.08 | 2.88E-10 |
| BC018222 | NM_144936.1 | 3.76 | 3.73E-09 |
| 2300005B03Rik | XM_128173.2 | 3.58 | 1.32E-09 |
| Cidea | NM_007702.1 | 3.56 | 6.51E-10 |
| LOC208963 | XM_148065.2 | 3.54 | 2.21E-09 |
| Krtap16-7 | NM_130875 | 3.41 | 2.99E-08 |
| Aldh3a1 | NM_007436.1 | 3.33 | 5.73E-08 |
| Krt2-6b | NM_010669.1 | 3.28 | 6.80E-09 |
| Igk-C | XM_132633.4 | 3.21 | 8.68E-10 |
| Elovl6 | NM_130450.1 | 3.18 | 1.81E-08 |
| Fa2h | NM_178086.2 | 3.17 | 4.03E-09 |
| Defb6 | NM_054074.1 | 3.14 | 1.84E-06 |
| Serpina1b | NM_009244.2 | 3.06 | 8.17E-11 |
| Serpinb12 | NM_027971.1 | 3.01 | 1.45E-09 |
| Gpx2 | NM_030677.1 | 3.01 | 7.38E-10 |
| 5430420C16Rik | NM_175165.2 | 2.97 | 1.13E-09 |
| C1qtnf3 | NM_030888.2 | 2.96 | 7.22E-09 |
| Mfap4 | NM_029568.1 | 2.96 | 2.71E-09 |
| BC021614 | NM_144869.1 | 2.92 | 1.50E-09 |
| Mmp13 | NM_008607.1 | 2.89 | 7.94E-10 |
| Clra | NM_153506 | 2.85 | 5.79E-08 |
| LOC232925 | XM_149891.1 | 2.85 | 8.99E-11 |
| Krt2-6b | NM_010669.1 | 2.80 | 2.07E-08 |
| Sprr4 | NM_173070.1 | 2.75 | 1.47E-09 |
| Timp1 | NM_011593 | 2.73 | 9.31E-09 |
| Cyp17a1 | NM_007809.2 | 2.73 | 1.33E-07 |
| Ybx3 | AK029441 | 2.69 | 2.27E-05 |
| BC031593 | NM_146063.1 | 2.68 | 6.84E-06 |
| Igh-VJ558 | XM_354700 | 2.67 | 5.24E-09 |
| Krt1-16 | NM_008470.1 | 2.61 | 1.63E-05 |
| Defb4 | NM_019728.2 | 2.59 | 1.20E-09 |
| Nppb | NM_008726.2 | 2.58 | 1.41E-07 |
| 2410039E07Rik | NM_212483 | 2.57 | 4.28E-10 |
| Sprr2d | NM_011470.1 | 2.56 | 1.09E-08 |
| Acox2 | NM_053115.1 | 2.54 | 1.39E-07 |
| Krt2-6a | NM_008476.2 | 2.50 | 6.22E-09 |
| Rptn | NM_009100.1 | 2.49 | 9.40E-09 |
| Serpina1b | NM_009244.2 | 2.44 | 1.09E-08 |
| Cxcl16 | NM_023158.3 | 2.39 | 2.64E-10 |
| Mcpt4 | NM_010779 | 2.38 | 7.50E-10 |
| Igk-C | XM_132633.4 | 2.38 | 3.26E-08 |
| Epgn | NM_053087.1 | 2.34 | 1.76E-07 |
| Myl2 | NM_010861 | 2.34 | 7.10E-08 |
| Cxcl1 | NM_008176.1 | 2.31 | 3.04E-09 |
| Panx3 | NM_172454.1 | 2.31 | 1.80E-06 |
| Hp | NM_017370.1 | 2.28 | 2.65E-07 |
| LOC224046 | XM_147198.3 | 2.28 | 1.61E-07 |
| Egln3 | NM_028133.1 | 2.27 | 1.25E-08 |
| Ier3 | NM_133662.1 | 2.26 | 9.34E-07 |
| Qscn6 | NM_023268 | 2.24 | 1.88E-07 |
| Klk10 | NM_133712.1 | 2.21 | 3.30E-06 |
| Stat3 | NM_011486.2 | 2.20 | 1.47E-07 |
| Rbp1 | NM_011254.2 | 2.20 | 7.67E-08 |
| C79267 | NM_183148.1 | 2.19 | 2.16E-08 |
| Dhcr24 | NM_053272 | 2.19 | 7.78E-08 |
| 2510049J12Rik | XM_132808.1 | 2.17 | 2.27E-07 |
| Rarres1 | XM_130987.3 | 2.17 | 7.22E-09 |
| Prss22 | NM_133731.1 | 2.14 | 3.84E-07 |
| Matn4 | NM_013592.2 | 2.14 | 2.73E-06 |
| Igl-V1 | XM_148393.1 | 2.13 | 1.74E-07 |
| 1110055O21Rik | AK027978 | 2.12 | 6.60E-09 |
| Crabp2 | NM_007759.1 | 2.11 | 7.27E-07 |
| Thrsp | NM_009381.2 | 2.11 | 9.56E-07 |
| Cxcl9 | NM_008599.1 | 2.10 | 2.63E-06 |
| Car12 | NM_178396 | 2.09 | 2.48E-08 |
| Bcl11b | NM_021399.1 | 2.09 | 1.65E-07 |
| Saa1 | NM_009117.1 | 2.09 | 3.76E-08 |
| 2210420J11Rik |  | 2.06 | 2.84E-08 |
| Ces3 | NM_053200.1 | 2.05 | 2.97E-08 |
| Mgll | NM_011844.3 | 2.04 | 5.88E-08 |
| BC054059 | NM_145635.1 | 2.04 | 1.43E-07 |
| Mcptl | NM_008573 | 2.04 | 9.15E-08 |
| 1190026I17Rik | AK028032 | 2.03 | 1.12E-08 |
| 2510002J07Rik | AK002422 | 2.03 | 2.13E-07 |
| Acas2 | NM_019811.2 | 2.03 | 4.52E-08 |
| Gch1 | NM_008102.2 | 2.01 | 3.45E-06 |
| F2r | NM_010169.2 | 2.01 | 2.32E-08 |
| Casp1 | NM_009807.1 | 2.00 | 1.24E-08 |
