# Supplementary material for: Loss of Grainy Head-Like 1 Is Associated with Disruption of the Epidermal Barrier and Squamous Cell Carcinoma of the Skin
Source: PLoS One. 2014 Feb 20;9(2):e89247. doi: 10.1371/journal.pone.0089247 (PMC3930704; doi:10.1371/journal.pone.0089247)
Supplement: Table S3 — List of primers used in Q-RT-PCR. The abbreviations are: R – reverse, F – forward. (DOC) [file pone.0089247.s004.doc]

**Table S3.** List of primers used in Q-RT-PCR. The abbreviations are: R – reverse, F – forward.

| primer name | primer sequence | source |
| --- | --- | --- |
| S100A9-R | 5'-GCTCAGCTGATTGTCCTGGT-3' | [16] |
| S100A9-F | 5'-TCAGACAAATGGTGGAAGCA-3' |
| S100A8-R | 5'-GTGGTAGACATCAATGAGGTTGCT-3' | kindly provided by Agnieszka Graczyk |
| S100A8-F | 5'-ATGCCGTCTGAACTGGAGAAG-3' |
| HPRT-R | 5'-CACAGGACTAGAACACCTGC-3' | [8] |
| HPRT-F | 5'-GCTGGTGAAAAGGACCTCT-3' |
