# Supplementary material for: Loss of Grainy Head-Like 1 Is Associated with Disruption of the Epidermal Barrier and Squamous Cell Carcinoma of the Skin
Source: PLoS One. 2014 Feb 20;9(2):e89247. doi: 10.1371/journal.pone.0089247 (PMC3930704; doi:10.1371/journal.pone.0089247)
Supplement: Materials and Methods S1 — Spontaneous cancers development. Microarray analysis of Grhl1 +/+ and Grhl1 −/− mouse skin. (DOC) [file pone.0089247.s005.doc]

**Supplementary materials and methods**

Spontaneous cancers development

Mice (n=13 per genotype) were kept in the Nencki Institute Animal House under standard conditions and under weekly observation. After 30 months the remaining animals were dissected and visually analyzed for irregularities.

Microarray analysis of *Grhl1*^+/+^ and *Grhl1*^-/-^ mouse skin

The expressional microarray on RNA isolated from whole skin had been performed earlier [9]. The obtained data were analyzed and the hits with significantly increased expression (at least doubled expression; p<0.05) are listed in Table S2. From this group the 69 specific genes were taken for further analysis. In this group about 60% of genes have been linked to the inflammation process or are associated with functions of the immune system (Table 1).
